# RETRACTION: Effects of Predictive Nursing Interventions on Pressure Ulcer in Elderly Bedridden Patients

**DOI:** 10.1111/iwj.70149

**Published:** 2024-12-05

## Abstract

**RETRACTION:** DengG.‐L. , LeiY.‐L. , TanH. , GengB.‐C. , and LiuZ. , “Effects of Predictive Nursing Interventions on Pressure Ulcer in Elderly Bedridden Patients,” International Wound Journal 21, no. 3 (2024): e14690, 10.1111/iwj.14690.38453139 PMC10920028

The above article, published online on 07 March 2024, in Wiley Online Library (http://onlinelibrary.wiley.com/), has been retracted by agreement between the journal Editor in Chief, Professor Keith Harding; and John Wiley & Sons Ltd. Following an investigation by the publisher, all parties have concluded that this article was accepted solely on the basis of a compromised peer review process. In addition, the authors provided incomplete ethical approval and patient consent information for the study. The editors have therefore decided to retract the article. The authors did not respond to our notice regarding the retraction.

**RETRACTION:** G.‐L. Deng , Y.‐L. Lei , H. Tan , B.‐C. Geng , and Z. Liu , “Effects of Predictive Nursing Interventions on Pressure Ulcer in Elderly Bedridden Patients,” International Wound Journal 21, no. 3 (2024): e14690, 10.1111/iwj.14690.38453139 PMC10920028

The above article, published online on 07 March 2024, in Wiley Online Library (http://onlinelibrary.wiley.com/), has been retracted by agreement between the journal Editor in Chief, Professor Keith Harding; and John Wiley & Sons Ltd. Following an investigation by the publisher, all parties have concluded that this article was accepted solely on the basis of a compromised peer review process. In addition, the authors provided incomplete ethical approval and patient consent information for the study. The editors have therefore decided to retract the article. The authors did not respond to our notice regarding the retraction.

